# Epithelial Cells Attenuate Toll-Like Receptor-Mediated Inflammatory Responses in Monocyte-Derived Macrophage-Like Cells to *Mycobacterium tuberculosis* by Modulating the PI3K/Akt/mTOR Signaling Pathway

**DOI:** 10.1155/2018/3685948

**Published:** 2018-09-26

**Authors:** Yi Yang, Yingfei Sun, Jinrui Xu, Kangda Bao, Meihui Luo, Xiaoming Liu, Yujiong Wang

**Affiliations:** ^1^Key Laboratory of Ministry of Education for Conservation and Utilization of Special Biological Resources in Western China, Ningxia University, Yinchuan, Ningxia 750021, China; ^2^College of Life Science, Ningxia University, Yinchuan, Ningxia 750021, China

## Abstract

Both alveolar macrophages (AMs) and alveolar epithelial cells (AECs) are main targets of *Mycobacterium tuberculosis* (*M. tuberculosis* (*Mtb*)). Intercellular communications between mucosal AECs and AMs have important implications in cellular responses to exogenous insults. However, molecular mechanisms underpinning interactions responding to *Mtb* remain largely unknown. In this study, impacts of AECs on Toll-like receptor- (TLR-) mediated inflammatory responses of AMs to *Mtb* virulent strain H37Rv were interrogated using an air-liquid interface (ALI) coculture model of epithelial A549 cells and U937 monocyte-derived macrophage-like cells. Results showed that *Mtb*-activated TLR-mediated inflammatory responses in U937 cells were significantly alleviated when A549 cells were coinfected with H37Rv, in comparison with the infection of U937 cells alone. Mechanistically, PI3K/Akt/mTOR signaling was involved in the epithelial cell-modulated *Mtb*-activated TLR signaling. The epithelial cell-attenuated TLR signaling in U937s could be reversed by PI3K inhibitor LY294002 and mTOR inhibitor rapamycin, but not glycogen synthase kinase 3*β* inhibitor LiCl, suggesting that the epithelially modulated-TLR signaling in macrophages was in part caused by inhibiting the TLR-triggered PI3K/Akt/mTOR signaling pathway. Together, this study demonstrates that mucosal AEC-derived signals play an important role in modulating inflammatory responses of AMs to *Mtb*, which thus also offers an insight into cellular communications between AECs and AMs to *Mtb* infections.

## 1. Introduction

Tuberculosis remains a global threat due to the emergence of drug-resistant *Mycobacterium tuberculosis* (*Mtb*) strains and coinfection to HIV; a better understanding of the mechanisms that mediate protection and pathogenesis of this organism is therefore crucial for improving strategies to control and reduce tuberculosis burdens worldwide [1]. Epidemiologically, the deposition of bacilli into alveolar sacs is the most common route of *Mtb* infection following an inhalation. In this regard, alveolar macrophages and dendritic cells have been recognized as key players in the establishment of host responses during an *Mtb* infection. In addition, alveolar epithelial cells (AECs) are the dominant cell type in alveolar sacs; the role of AECs in host defense of *Mtb* infection however has not been fully appreciated until the recently emerging evidence that AECs were also host targets of *Mtb* has been discovered. Apart from their function as epithelial barriers, AECs could also exert immunoregulatory roles as mucosal nonprofessional immune cells in response to infections [2–5].

In this respect, a compelling body of evidence demonstrated that AECs acted as a bridge for the communication between innate and adaptive immune systems to initiate and shape immune responses in the lungs [2–5]. Functionally, AECs were able to internalize *Mtb* bacterial cells and present antigens to primed T cells or acted as a reservoir of pathogens. In addition, AECs also were capable of secreting soluble mediators, such as cytokines and chemokines upon an infection, although the profile and pattern of secretions were different between AECs and alveolar macrophages [3].

Importantly, an increased number of studies recently demonstrated that AECs were able to modulate the immune response of alveolar macrophages in a contact-dependent or contact-independent manner in the lungs. Indeed, the immunoregulatory effects of AECs on immune responses of macrophages to various stimuli have recently been reported from different groups. An interaction between epithelial cells and macrophages might modulate and/or shape phenotypes of macrophages in response to an insult [6–10]. For instance, rat RLE-6TN cells or human A549 cells exhibited an ability to reduce LPS-induced nitric oxide (NO) production, accompanied by a decreased expression of inducible NO synthase (iNOS) and interleukin-6 (IL-6) in alveolar macrophages in a contact-independent mechanism [9]. In another study, Stříž et al. examined the effect of epithelial cells on macrophages and found that epithelial cells could augment intercellular adhesion molecule 1 (ICAM-1 or CD54) and HLA-DR in THP-1 monocytes in a cell-cell contact-dependent manner. The enhanced effects were diminished when the cells were separated by a filter insert, suggesting an importance of epithelial cells in regulating the phenotype of human macrophages [6].

Of interest, the effect of epithelial cells on immune responses of macrophages was in a pathogen- (or stimulus-) dependent manner. For examples, in one study, alveolar epithelial type II (AEC II) cells were demonstrated to enhance immune response (cytokine release) of macrophages in response to stimuli of various particles [7]; however, an opposite effect was reported in another study, i.e., AECs could alleviate cytokine release of alveolar macrophages in response to JP-8 jet fuel [8]. Such an inhibitory role of AECs in modulating inflammatory responses of alveolar macrophages was also reported in a study where rat RLE-6TN or human A549 cells were stimulated with LPS [9]. These findings clearly indicated an importance of AECs in shaping immune phenotypes of alveolar macrophages in response to an infection of various pathogens, including *Mtb*.

In order to better understand the role of signaling of AECs in modulating inflammatory responses of alveolar macrophages to an *Mtb* infection, the impact and molecular mechanisms of AECs on modulating Toll-like receptor- (TLR-) triggered inflammatory responses of alveolar macrophages to *Mtb* were interrogated in a contact-independent coculture model of A549 epithelial cells and U937 mononuclear cell-derived macrophage-like cells in the present study.

## 2. Material and Methods

### 2.1. Cell Lines

The human leukemic monocyte U937 cell line and human lung adenocarcinoma epithelial A549 cell line were purchased from American Type Culture Collection (ATCC, Manassas, VA, USA). Cells were maintained in RPMI 1640 supplemented with 10% fetal bovine serum (FBS) (Invitrogen, Carlsbad, CA, USA) and penicillin/streptomycin (Sigma, St. Louis, MO, USA). Cells were cultured in humidified incubators with 5% CO_2_ atmosphere at 37°C.

### 2.2. *M. tuberculosis* H37Rv Culture

*M. tuberculosis* virulent strain H37Rv was provided from China Institute of Veterinary Drug Control (Beijing, China). All experiments related to *M. tuberculosis* H37Rv strain were conducted in a Biosafety Level III (BSL III) laboratory. Mtb H37Rv bacterial cells were grown in Middlebrook 7H9 medium (BD Difco) supplemented with 10% ADC (Difco™ BD Bioscience, San Jose, CA, USA) and 0.05% Tween 80 (Sigma, St. Louis, MO, USA) at 37°C. Cells were harvested at the mid-log phase and were pelleted and resuspended in RPMI-1640 at a concentration of 10^8^ bacilli/mL. The cells were then aliquoted and stored at −80°C as working stocks till use.

### 2.3. Mtb Infection of the Coculture Model of Epithelial A549 Cells and U937 Cell-Derived Macrophages

**Figure 1:**
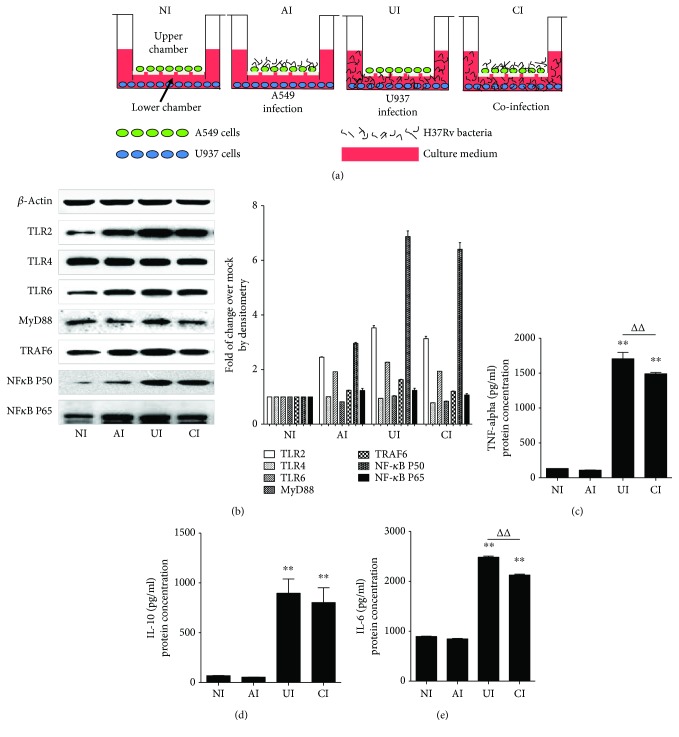
*Mtb* H37Rv-infected A549 cells reduced TLR signaling activity and cytokine production of U937 cells in response to mycobacterial infection. (a) Illustration of cell culture models used for infection in this study. A549 cells were cultured on the apical surface of transwells at an air-liquid interface state for 24 hours; then the transwell insert was transferred to a well containing PMA-stimulated U937 cells for infection. The coculture model of A549/U937 cells was infected with H37Rv mycobacteria from the upper chamber (A549 cells, AI), lower chamber (U937 cells, UI), or both chambers (A549 and U937 cells, CI) at a MOI of 3 for 18 h before the culture medium and U937 cells were harvested for analysis. (b) Representative blots of immunoblotting assay for indicated components of TLR signaling cascade (left panel) and fold of changes of proteins of interest in U937 cells semiquantitatively determined by densitometric assay using ImageJ software Fiji (right panel). H37Rv-infected A549 cells showed an ability to reduce TLR signaling activity in U937 cells in response to mycobacterial infection. Concentrations of TNF-*α* (c), IL-10 (d), and IL-6 (e) in culture media determined by ELISA. H37Rv-infected A549 cells led a reduction of cytokine production in U937 cells in response to *Mycobacteria* infection. Error bars represent the standard deviation (SD) from three independent experiments. Compared to noninfection (NI) control, ^∗∗^ *p* < 0.01; compared to infection of U937 cell alone, ^ΔΔ^ *p* < 0.01. NI: noninfected control; AI: infection was performed on A549 cell alone; UI: infection was performed on U937 alone; CI: infection was performed on both A549 cells and U937 cells.

To generate a cell contact-independent model, the epithelial A549 cells were seeded on the upper chamber of a transwell (24 mm diameter transwell with 0.4 *μ*m pore polyester membrane insert, 3450 Clear, Corning Life Sciences, Corning, NY, USA) at a density of 5 × 10^5^ cells/insert in culture medium and cultured overnight; the medium of the upper chamber was subsequently removed and the cells were cultured for an additional 24 hours to establish an air-liquid interface (ALI). At the time of seeding of A549 cells, 5 × 10^6^ cells/well of U937 cells were separately cultured in another 6-well plate (Primera; Becton-Dickinson Labware, Franklin Lakes, NJ, USA) in RPMI 1640 medium supplemented with 10% FBS containing 0.2 *μ*g/mL phorbol-12-myristate-13-acetate (PMA) to derive macrophages overnight. The cells were then refreshed with regular culture medium and cultured for an additional 24 h before the establishment of the coculture model. The above ALI A549 cell culture was then placed into the well of 6-well plates containing U937 cell-derived macrophages that were independently pretreated with PMA to assemble the epithelial cell/macrophage coculture model. The cocultures were subsequently infected with *Mtb* H37Rv at a multiplicity of infection (MOI) of 3 in culture medium for 18 h (Figure 1(a)). The infection was conducted for A549 cells (upper chamber) or U937 macrophages (lower chamber) alone, or coinfection of A549 cells and U937 cells from both the upper and lower chambers (Figure 1(a)). The culture medium, A549 cells, and U937 cell-derived macrophages were harvested for analysis.

### 2.4. RNA Isolation and Real-Time Quantitative PCR

The total RNA from different treated cells was isolated using TRIzol reagent as per the manufacturer's instruction (Invitrogen, Grand Island, NY, USA). The quality of RNA was assayed by calculating the RNA integrity number (RIN) [11]. High-quality RNA (RIN value was greater than 9.0) was used for reverse transcription of first-strand cDNA synthesis by reverse transcription using M-MLV reverse transcriptase (Takara, Dalian, China). The quantitative real-time RT-PCR (qRT-PCR) was performed in the Roche LightCycler 2.0 using the Takara SYBR Green I kit (Takara, Dalian, China); the thermal cycling condition for PCR amplification was 95°C for 30 s, 40 cycles of 95°C for 5 s, 60°C for 20 s, and 72°C for 20 s, followed by 40°C for 20 min. The primer sets used for RT-PCR were designed and synthesized in Shanghai Sangon Biotech Inc. (Shanghai, China) by bioinformatics tools using available mRNA sequences (Table 1). The controls were always included to normalize each reaction with respect to RNA integrity, sample loading, and inter-PCR variations. The relative expression ratio was calculated from the real-time PCR efficiencies and the crossing point deviation of a given gene *vs β*-actin endogenous control. In each independent experiment, the mean gene expression ratios obtained with uninfected cultures were given a value of 1 (fold). Fold changes over controls were calculated by a 2^−ΔΔCT^ method to determine mRNA expression levels [12]. The specificity of PCR was determined by sequencing of the PCR products.

### 2.5. Western Blot Analysis

Whole cell extracts were prepared by homogenizing cells in lysis buffer (50 mM Tris-HCl, pH 7.5, 5 mM EDTA, 150 mM NaCl, 0.5% NP-40) for 60 min on ice. The soluble protein concentration was measured with Bio-Rad protein assay (Bio-Rad Laboratories, Richmond, CA, USA). The resulting clarified lysates (20–40 *μ*g) were resolved by 10% sodium dodecyl sulfate (SDS) polyacrylamide gel (SDS-PAGE) and transferred to nitrocellulose membranes for immunoblotting assay. The membrane was blocked with 5% fat-free dry milk in PBS containing 0.2% Tween 20 and probed with antibodies against proteins of interest. The antibodies of rabbit anti-Akt, rabbit anti-glycogen synthase kinase 3*β* (GSK3*β*), and rabbit anti-mTOR were products of Proteintech Group Inc. (Proteintech Group, Wuhan, China); antibodies rabbit anti-TRAF6, rabbit anti-MyD88, and rabbit anti-TLR-2 were purchased from Cell Signaling Technology (Cell Signaling Technology, Danvers, MA, USA); antibodies rabbit anti-TLR-4, rabbit anti-NF-*κ*B-p50, and rabbit anti-NF-*κ*B-p65 were products of Santa Cruz Biotechnology (Santa Cruz Biotechnology, Santa Cruz, CA, USA); rabbit anti-pAkt, rabbit anti-pGSK3*β*, and rabbit anti-pmTOR were purchased from Signalway Antibody (Signalway Antibody, College Park, MD, USA); antibody of rabbit anti-TLR6 was a product of Abcam (Abcam, Cambridge, MA, USA). The blot was washed, exposed to appropriately respective HRP-labeled secondary antibodies. The abundance of protein of interest was quantified by optical densitometry using ImageJ Software Fiji (https://imagej.net/Using_Fiji). The ratio between the net intensities of each sample divided by the *β*-actin internal control was calculated as densitometric arbitrary unit (A.U.) and served as an index of relative expression of a protein of interest. Fold change was calculated as the ratio between the net intensity of each sample divided by the respective internal controls (*β*-actin) [13].

### 2.6. Enzyme-Linked Immunosorbent Assay (ELISA)

The concentration of cytokines IL-6, IL-10, and TNF-*α* was determined using commercially available enzyme-linked immunosorbent assay (ELISA) kits as per the manufacturer's instructions (ProZyme, Hayward, CA, USA). All spectrophotometric readings were performed with a microplate reader, and the concentration was presented as pg/mL by comparing with respective standard curves. All experiments were repeated at least three times.

### 2.7. Statistical Analysis

All data in this study were presented as the mean ± SD of data from at least three independent experiments. SPSS statistics 19.0 (SPSS Inc., Chicago, IL, USA) was used for the statistical analysis. Statistical differences between groups were performed using one-way analysis of variance (ANOVA) followed by post hoc Tukey's test. *p* values < 0.05 were considered statistically significant.

## 3. Results

### 3.1. Mtb-Infected A549 Cells Attenuated the Pathogen-Triggered TLR-Mediated Inflammatory Responses in U937 Monocyte-Derived Macrophage-Like Cells

**Figure 2:**
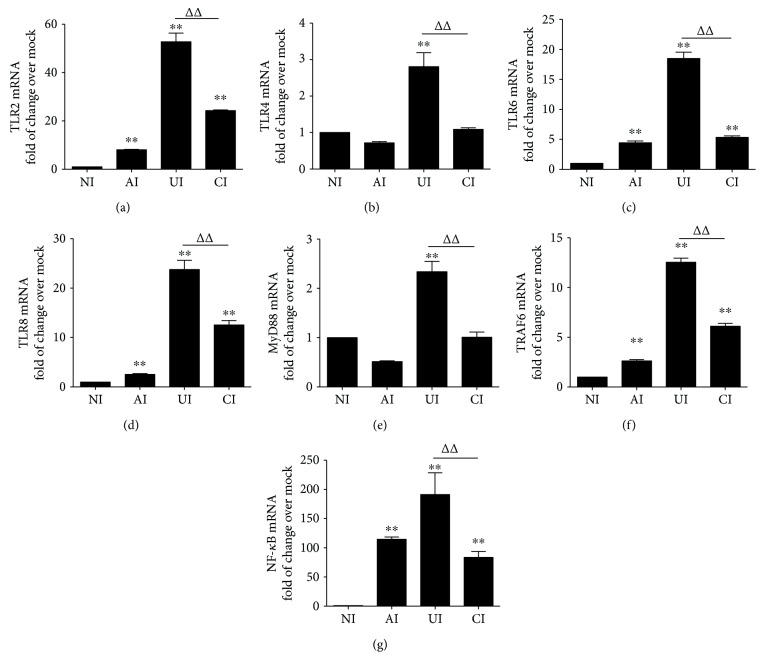
*Mtb* H37Rv-infected A549 cells inhibited the expression of TLR signaling of U937 cells in response to *Mycobacteria* infection. The coculture model of A549/U937 cells was infected with H37Rv *Mycobacteria* from the upper chamber (A549 cells, AI), lower chamber (U937 cells, UI), or both chambers (A549 and U937 cells, CI) at a MOI of 3 for 18 h before the U937 cells were harvested for analysis by RT-PCR assay. (a–g) Inductions of indicated transcripts of U937 cells infected with H37Rv in different conditions. (a) Fold of changes of TLR-2 transcripts over the noninfected cells; (B) fold of changes of TLR-4 transcripts over the noninfected cells; (c) fold of changes of TLR-6 transcripts over the noninfected cells; (d) fold of changes of TLR-8 transcripts over the noninfected cells; (e) fold of changes of MyD88 transcripts over the noninfected cells; (f) fold of changes of TRAF6 transcripts over the noninfected cells; (g) fold of changes of NF-*κ*B transcripts over the noninfected cells. Error bars represent the standard deviation (SD) from three independent experiments. Compared to noninfection (NI) control, ^∗∗^ *p* < 0.01; compared to infection of U937 cell alone, ^ΔΔ^ *p* < 0.01. NI: noninfected control; AI: infection was performed on A549 cell alone; UI: infection was performed on U937 alone; CI: infection was performed on both A549 cells and U937 cells.

**Figure 3:**
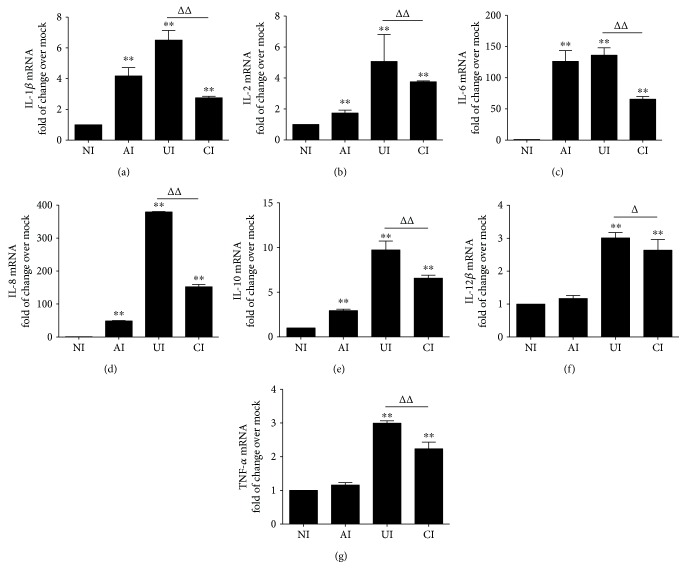
*Mtb* H37Rv-infected A549 cells reduced the expression of cytokines of U937 cells in response to mycobacterial infection. The coculture model of A549/U937 cells was infected with H37Rv mycobacteria from the upper chamber (A549 cells, AI), lower chamber (U937 cells, UI), or both chambers (A549 and U937 cells, CI) at a MOI of 3 for 18 h before the U937 cells were harvested for analysis by a RT-PCR assay. (a–g) Inductions of indicated transcripts of U937 cells infected with H37Rv in different conditions. (a) Fold of changes of IL-1*β* transcripts over the noninfected cells; (b) fold of changes of IL-2 transcripts over the noninfected cells; (c) fold of changes of IL-6 transcripts over the noninfected cells; (d) fold of changes of IL-8 transcripts over the noninfected cells; (e) fold of changes of IL-10 transcripts over the noninfected cells; (f) fold of changes of IL-12*β* transcripts over the noninfected cells; (g) fold of changes of TNF-*α* transcripts over the noninfected cells. Error bars represent the standard deviation (SD) from three independent experiments. Compared to noninfection (NI) control, ^∗∗^ *p* < 0.01; compared to infection of U937 cell alone, ^ΔΔ^ *p* < 0.01. NI: noninfected control; AI: infection was performed on A549 cell alone; UI: infection was performed on U937 alone; CI: infection was performed on both A549 cells and U937 cells.

In order to investigate the function of alveolar epithelial cells in modulating immune responses of macrophages to *Mtb* infections, a coculture model of epithelial A549 cells and PMA-induced U937 monocyte-derived macrophages was employed. The cells were infected with H37Rv mycobacteria from the upper chamber (A549 cells) or the lower chamber (U937 cells) alone, or from both sides (A549 and U937 cells), and the expression of TLR ligands and elements of the TLR signaling pathway in U937 cells were analyzed (Figure 1(a)). Results of immunoblotting assay showed an increased expression of TLR-2 and TLR-6, TRAF-6, and NF-*κ*B proteins in U937 cells in all three infected conditions (i.e., either infected with A549 cells or U937 cells alone or coinfected with both cell types) as compared with uninfected cells in the coculture model (Figure 1(b)). Surprisingly, the abundance of TLR-4 was not altered and an increased expression of MyD88 protein was only observed in H37Rv-infected U937 cells alone (Figure 1(b)). However, a robust production of cytokines TNF-*α* (Figure 1(c)), IL-10 (Figure 1(d)), and IL-6 (Figure 1(e)) was only observed in cocultures when U937 cells were infected with H37Rv, but not in those cocultures that A549 cells were infected alone, as determined by an ELISA (Figures 1(c)–1(e)). More importantly, the coinfection of H37Rv to A549 cells significantly reduced the expression of *Mtb*-triggered TLR signaling cascade of U937 cell-derived macrophages (Figure 1(b)) and the production of the examined cytokines, in comparison with U937 cell infection alone (*p* < 0.01) (Figures 1(c)–1(e)). Transcriptional analysis by RT-PCR assay further demonstrated an increased abundance of transcripts of TLR-signaling cascaded TLR-2 (Figure 2(a)), TLR-4 (Figure 2(b)), TLR-6 (Figure 2(c)), TLR-8 (Figure 2(d)), MyD88 (Figure 2(e)), TRAF-6 (Figure 2(f)), and NF-*κ*B (Figure 2(g)), as well as transcripts of cytokines IL-1*β* (Figure 3(a)), IL-2 (Figure 3(b)), IL-6 (Figure 3(c)), IL-8 (Figure 3(d)), IL-10 (Figure 3(e)), IL-12*β* (Figure 3(f)), and TNF-*α* (Figure 3(g)) in all three infected conditions of H37Rv (*p* < 0.01). Similar to that observed in immunoblotting assay and ELISA, a coinfection of *Mtb* H37Rv to A549 cells dramatically decreased the expression of the above TLR signaling components and cytokines at the transcriptional level of the H37Rv-infected U937 cells (*p* < 0.01). Of note, using transcriptional analysis of the A549 epithelial cells, the infection of *Mtb* H37Rv dramatically activated TLR signaling and cytokines in A549 cells as seen in the above U937 monocyte-derived macrophages (supplementary data Figure 2 and Figure 2). However, the coinfection also significantly reduced the expression of TLR ligands and signaling cascades TLR-4, TLR-6, TLR-8, MyD88, TRAF-6, and NF-*κ*B (supplementary data Figure 1), as well as transcripts of cytokines IL-1*β*, IL-2, IL-6, IL-8, and IL-10 (Figures 3(a)–3(e)), but not IL-12*β* and TNF-*α* (supplementary data Figure 2), as compared with infection of U937 cell-derived macrophage cells alone (*p* < 0.01). Even more interestingly, the infection of U937 cells alone showed the most robust induction of transcripts of TLR signaling elements and cytokines in U937 cells (supplementary data Figure 1 and Figure 2). These data imply that a coinfection of Mtb H37Rv to epithelial A549 cells has an ability to reduce the expression of TLR ligands and elements of the TLR signaling pathway in macrophages to an *Mtb* infection.

### 3.2. PI3K/Akt Signaling Is Involved in the Modulation of Epithelial A549 Cells to Inflammatory Responses of U937 Monocyte-Derived Macrophage-Like Cells to Mtb

**Figure 4:**
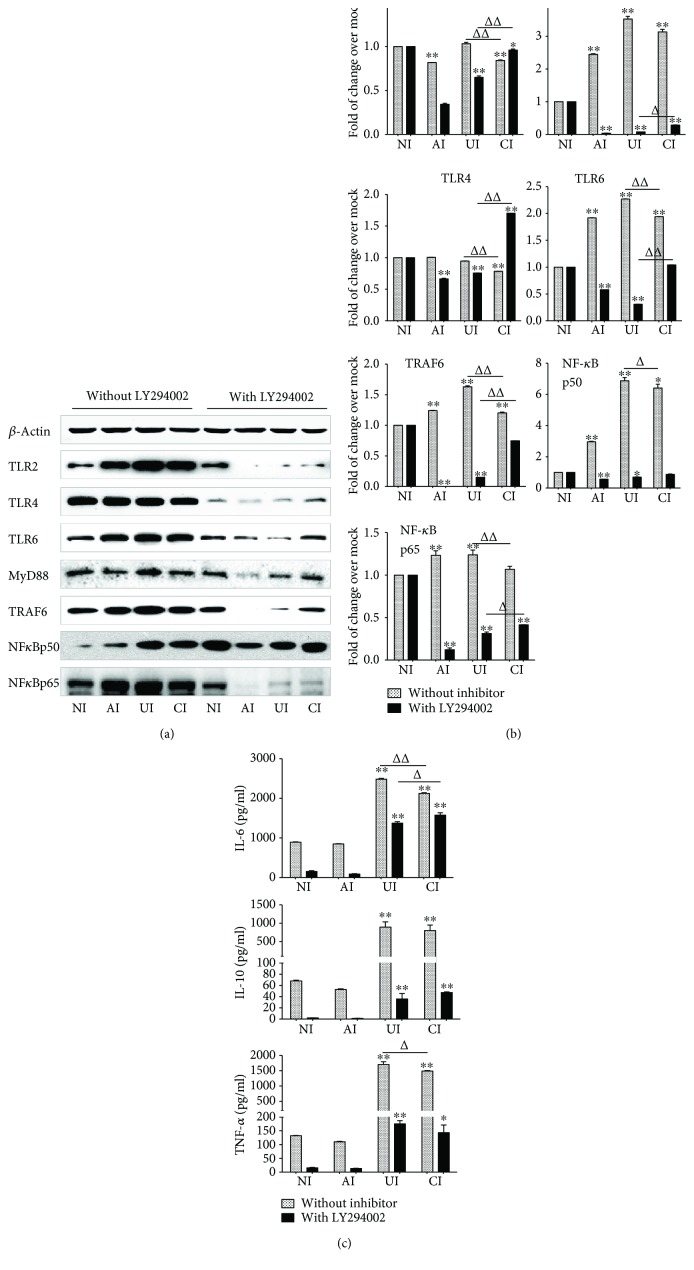
Involvement of PI3K signaling in the reduction of the expression of TLR ligands and elements of TLR signaling pathway in U937 cells to *Mtb* H37Rv infection in the A549 cell coculture model. In the presence or absence of PI3K inhibitor LY294002, the coculture model of A549/U937 cells was infected with H37Rv mycobacteria from the upper chamber (A549 cells, AI), lower chamber (U937 cells, UI), or both chambers (A549 and U937 cells, CI) at a MOI of 3 for 18 h before the culture medium and U937 cells were harvested for analysis. (a) Representative blots of immunoblotting assay for the indicated components of TLR signaling cascade showed a reversed TLR signaling activity in U937 cells of the coinfection model in the presence of LY294002, in comparison with the absence of an inhibitor. (b) The fold of changes of proteins of interest in (a) semiquantitatively determined by densitometric assay using ImageJ software Fiji from three independent experiments. The ability of A549 cell-mediated reduction of TLR signaling activity in U937 cells was reversed by the addition of LY294002. (c) Concentrations of TNF-*α*, IL-10, and IL-6 in culture media determined by ELISA; the A549 cell-mediated reduction of cytokines in H37Rv-infected U937 cells was reversed in the presence of PI3K inhibitor. Error bars represent the standard deviation (SD) from three independent experiments. Compared to noninfection (NI) control, ^∗^ *p* < 0.05 and ^∗∗^ *p* < 0.01; compared to the absence of LY294002, ^Δ^ *p* < 0.05 and ^ΔΔ^ *p* < 0.01. NI: noninfected control; AI: infection was performed on A549 cell alone; UI: infection was performed on U937 alone; CI: infection was performed on both A549 cells and U937 cells.

**Figure 5:**
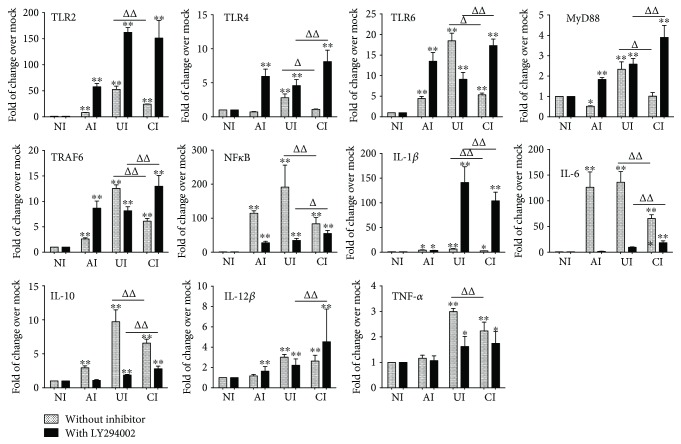
Impact of PI3K signaling in U937 cells in response to H37Rv infection. In the presence or absence of PI3K inhibitor LY294002, the coculture model of A549/U937 cells was infected with H37Rv mycobacteria from the upper chamber (A549 cells, AI), lower chamber (U937 cells, UI), or both chambers (A549 and U937 cells, CI) at a MOI of 3 for 18 h before the culture medium and U937 cells were harvested for analysis by RT-PCR assay. Inductions of indicated transcripts of U937 cells infected with *Mtb* H37Rv in different conditions. The data was presented as the fold of changes of indicated transcripts over the noninfected cells. The ability of A549 cell-mediated reduction of TLR-mediated inflammations in U937 cells was reversed by the addition of LY294002. Error bars represent the standard deviation (SD) from three independent experiments. Compared to noninfection (NI) control, ^∗^ *p* < 0.05 and ^∗∗^ *p* < 0.01; compared to the absence of LY294002, ^Δ^ *p* < 0.05 and ^ΔΔ^ *p* < 0.01. NI: noninfected control; AI: infection was performed on A549 cell alone; UI: infection was performed on U937 alone; CI: infection was performed on both A549 cells and U937 cells.

**Figure 6:**
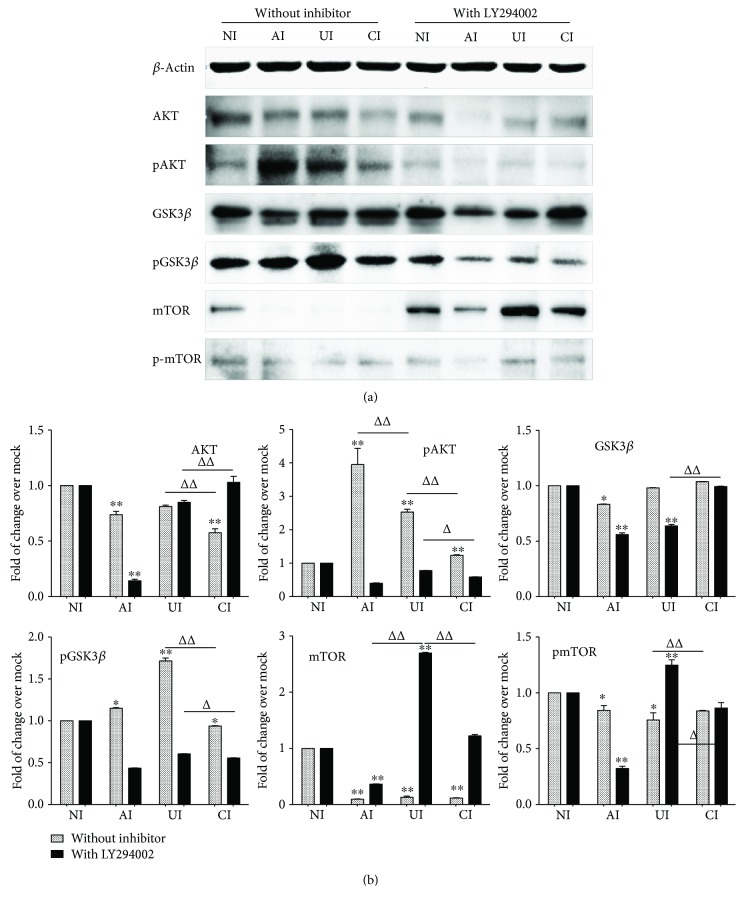
Impact of PI3K/Akt/GSK3*β*/mTOR signaling in U937 cells in response to H37Rv infection. In the presence or absence of PI3K inhibitor LY294002, the coculture model of A549/U937 cells was infected with H37Rv mycobacteria from the upper chamber (A549 cells, AI), lower chamber (U937 cells, UI), or both chambers (A549 and U937 cells, CI) at a MOI of 3 for 18 h before the culture medium and U937 cells were harvested for analysis. (a) Representative blots of immunoblotting assay for the indicated components of PI3K/Akt/GSK3*β*/mTOR signaling showed an involvement of Akt, GSK3*β*, and mTOR signaling in U937 cells of the coinfection model. (b) The fold of changes of proteins of interest in (a) semiquantitatively determined by densitometric assay using ImageJ software Fiji from three independent experiments; the ability of A549 cell-mediated reduction of Akt, GSK3*β*, and mTOR signaling activity in U937 cells was lost in the presence of LY294002. Error bars represent the standard deviation (SD) from three independent experiments. Compared to noninfection (NI) control, ^∗^ *p* < 0.05 and ^∗∗^ *p* < 0.01; compared to the absence of LY294002, ^Δ^ *p* < 0.05 and ^ΔΔ^ *p* < 0.01. NI: noninfected control; AI: infection was performed on A549 cell alone; UI: infection was performed on U937 alone; CI: infection was performed on both A549 cells and U937 cells.

Next, we sought to interrogate the possible molecular mechanism underpinning the immune regulatory roles of epithelial cells in inflammatory response of macrophages to *Mtb* infections. The phosphatidylinositol 3 kinase (PI3K)/Akt signaling is crucial for cell growth, proliferation, and differentiation. It has recently been demonstrated to play important roles in regulating immune responses in many cell types [14–16]; the involvement of PI3K/Akt signaling in the interaction between epithelial cells and macrophages therefore was examined. Intriguingly, the *Mtb* H37Rv-activated TLR signaling of U937-derived macrophage-like cells was almost completely abolished or inhibited by the addition of PI3K inhibitor LY294002 at final concentration of 100 *μ*M (Santa Cruz, Santa Cruz, CA, USA) as determined by an immunoblotting assay (Figures 4(a) and 4(b)). In addition, the *Mtb* H37Rv-induced production of IL-6, IL-10, and TNF-*α* was also significantly reduced in cells cultured in the presence of LY294002, as measured by an ELISA (*p* < 0.01) (Figure 4(c)). Equally noteworthy, the coinfection of A549 cell-attenuated TLR-signaling activity of *Mtb*-infected U937-derived macrophage-like cells and the Mtb-induced cytokine production were partially inhibited by the addition of LY294002 (*p* < 0.05 or *p* < 0.01) (Figure 4). The abrogation of A549 cell-mediated attenuation of TLR-signaling activity and cytokine production of *Mtb*-infected U937-derived macrophage-like cells by LY294002 was also supported by transcriptional analysis using RT-PCR assay, i.e., an addition of LY294002 reversed the H37Rv-infected A549 cell-reduced expression of TLR signaling and cytokines in the *Mtb*-infected U937-derived cells (*p* > 0.01) (Figure 5). As expected, an infection of Mtb H37Rv activated Akt and GSK3*β*, the downstream signaling of the PI3K pathway. More abundant phosphorylated Akt and GSK3*β* were detected in *Mtb*-infected U937 cells as determined by immunoblotting assay (Figure 6). Of note, a coinfection of H37Rv to A549 cells reduced the abundance of phosphorylated Akt and GSK3*β* in U937 macrophages (Figure 6). Interestingly, the infection of Mtb H37Rv did not alter the phosphorylation of mTOR, rather than reduce the expression of total mTOR protein in U937-derived macrophage-like cells (Figure 6). Even more noteworthy, the addition of PI3K inhibitor LY294002 led an abolished Mtb-induced phosphorylation of Akt and GSK3*β* but increased the expression of total mTOR protein in macrophages. The H37Rv-infected A549 cell-reduced phosphorylated Akt, GSK3*β*, and mTOR proteins were partially restored in U937-derived cells exposed to LY294002 (Figure 6). These data suggest that both PI3K/Akt/GSK3*β* signaling and PI3K/Akt/mTOR signaling may be involved in the modulation of epithelial cells to U937-derived macrophage-like cells in response to an *Mtb* infection.

### 3.3. The Impact of GSK3*β* Signaling on the Modulation of Epithelial A549 Cells to Mtb H37Rv-Induced Inflammatory Responses in U937 Monocyte-Derived Macrophage-Like Cells

**Figure 7:**
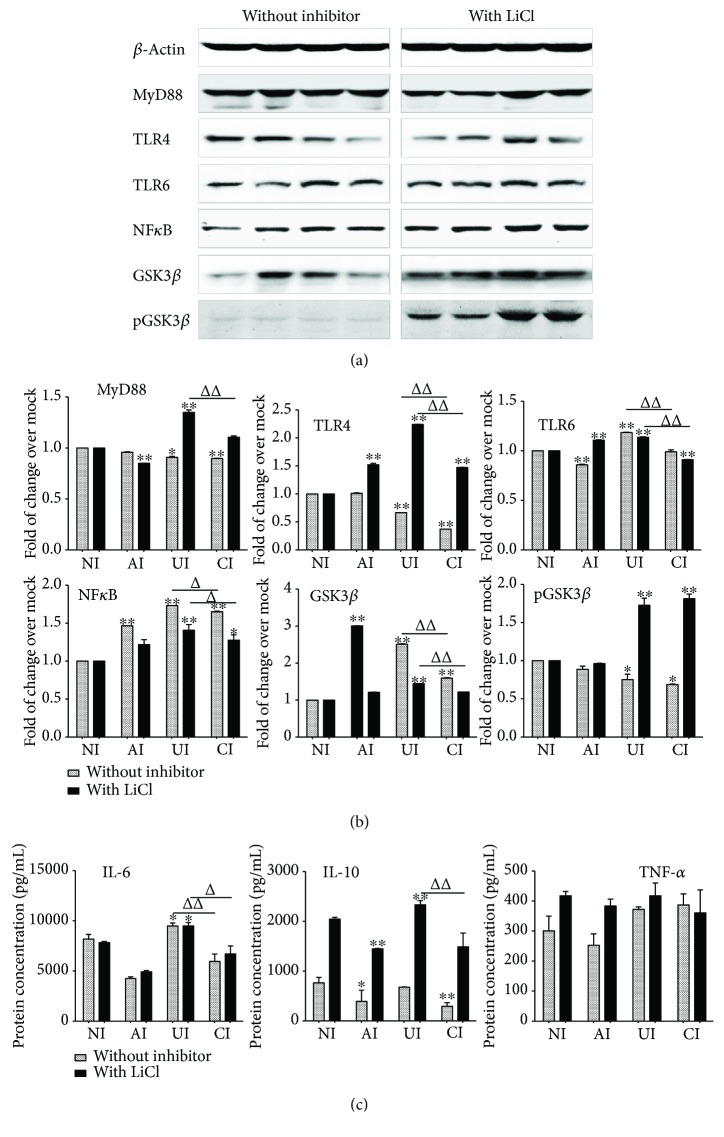
Impact of GSK3*β* on the expression of TLR ligands and elements of the TLR signaling pathway in U937 cells to Mtb H37Rv infection in the A549 cell coculture model. In the presence or absence of GSK3*β* inhibitor LiCl, the coculture model of A549/U937 cells was infected with H37Rv mycobacteria from the upper chamber (A549 cells, AI), lower chamber (U937 cells, UI), or both chambers (A549 and U937 cells, CI) at a MOI of 3 for 18 h before the culture medium and U937 cells were harvested for analysis. (a) Representative blots of immunoblotting assay for indicated components of TLR signaling cascade showed an activated TLR signaling in U937 cells of the coinfection model in the presence of LiCl, in comparison with the absence of an inhibitor. (b) The fold of changes of proteins of interest in (a) semiquantitatively determined by densitometric assay using ImageJ software Fiji from three independent experiments. (c) Concentrations of TNF-*α*, IL-10, and IL-6 in culture media determined by ELISA. An augmented cytokine production was observed in the presence of GSK3*β* inhibitor LiCl, but the trend of a reduced TLR-mediated inflammatory response was not altered by the addition of LiCl. Error bars represent the standard deviation (SD) from three independent experiments. Compared to noninfection (NI) control, ^∗^ *p* < 0.05 and ^∗∗^ *p* < 0.01; compared to the absence of LiCl, ^Δ^ *p* < 0.05 and ^ΔΔ^ *p* < 0.01. NI: noninfected control; AI: infection was performed on A549 cell alone; UI: infection was performed on U937 alone; CI: infection was performed on both A549 cells and U937 cells.

**Figure 8:**
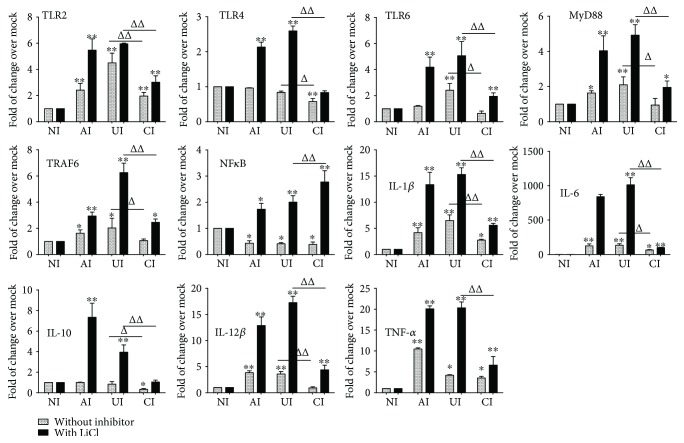
Impact of GSK3*β* signaling in U937 cells in response to H37Rv infection. In the presence or absence of GSK3*β* inhibitor LiCl, the coculture model of A549/U937 cells was infected with H37Rv mycobacteria from the upper chamber (A549 cells, AI), lower chamber (U937 cells, UI), or both chambers (A549 and U937 cells, CI) at a MOI of 3 for 18 h before the culture medium and U937 cells were harvested for analysis by RT-PCR assay. Inductions of indicated transcripts of U937 cells infected with H37Rv in different conditions. The data was presented as the fold of changes of indicated transcripts over the noninfected cells. An increased abundance of indicated TLR signaling and cytokines was observed in the presence of GSK3*β* inhibitor LiCl, but the trend of a reduction of TLR-mediated inflammatory responses was not altered by the addition of LiCl. Error bars represent the standard deviation (SD) from three independent experiments. Compared to noninfection (NI) control, ^∗^ *p* < 0.05 and ^∗∗^ *p* < 0.01; compared to the absence of LY294002, ^Δ^ *p* < 0.05 and ^ΔΔ^ *p* < 0.01. NI: noninfected control; AI: infection was performed on A549 cell alone; UI: infection was performed on U937 alone; CI: infection was performed on both A549 cells and U937 cells.

In order to further elaborate the role of PI3K/Akt/GSK3*β* in the interaction of epithelial cells and macrophages in response to *Mtb* infections, the TLR-mediated inflammatory responses of U937 macrophages were examined in the *Mtb* H37Rv-infected A549/U937-derived macrophage-like cell coculture model in the presence of GSK3*β* inhibitor LiCl. Immunoblotting assay showed that an exposure of A549/U937-cocultured cells to LiCl at final concentration of 10 mM (Sigma, St. Louis, MO, USA) led a robust phosphorylated GSK3*β* and a moderately increased expression of TLR4 and MyD88 proteins but reduced the abundance of NF-*κ*B and GSK3*β* proteins in U937-derived macrophage-like cells in response to the H37Rv infection (Figures 7(a) and 7(b)). Interestingly, the addition of LiCl did not alter the *Mtb*-induced production of cytokines IL-6 and TNF-*α* but significantly increased the IL-10 production as determined by an ELISA (*p* < 0.01) (Figure 7(c)). However, unlike that being seen in the presence of LY294002, the addition of LiCl failed to restore the A549 cell-reduced expression of TLR signaling elements and cytokine production in U937 cell-derived macrophage-like cells to *Mtb* (Figure 7). Transcriptional analysis further revealed the significantly increased transcripts of examined TLR signaling elements and cytokines in the macrophage-like cells of *Mtb*-infected cocultures in the presence of LiCl in comparison with those in the absence of LiCl (*p* < 0.01) (Figure 8). In agreement with the result of immunoblotting assay, the addition of LiCl could not restore the *Mtb*-infected epithelial cell-reduced transcripts of TLR-signaling molecules and cytokines, except the transcript of NF-*κ*B (*p* > 0.01) (Figure 8). These results imply that the A549 epithelial cell-attenuated activation of TLR signaling and cytokine production in U937-derived macrophage-like cells are not mediated by the PI3K/Akt/GSK3*β* signaling pathway.

### 3.4. The Impact of PI3K/Akt/mTOR Signaling on the Modulation of Epithelial A549 Cells to Mtb H37Rv-Induced Inflammatory Responses in U937 Monocyte-Derived Macrophage-Like Cells

**Figure 9:**
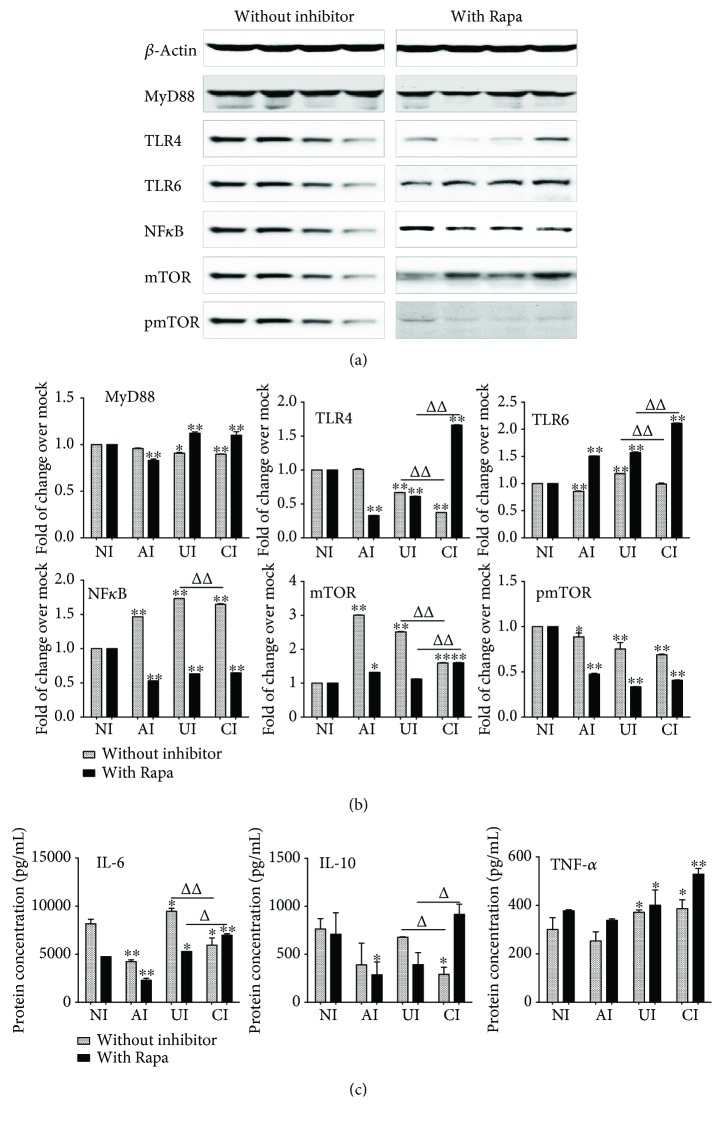
Impact of mTOR on the expression of TLR ligands and elements of the TLR signaling pathway in U937 cells to H37Rv infection in the A549 cell coculture model. In the presence or absence of mTOR inhibitor rapamycin, the coculture model of A549/U937 cells was infected with H37Rv mycobacteria from the upper chamber (A549 cells, AI), lower chamber (U937 cells, UI), or both chambers (A549 and U937 cells, CI) at a MOI of 3 for 18 h before the culture medium and U937 cells were harvested for analysis. (a) Representative blots of immunoblotting assay for indicated components of TLR signaling cascade showed an increased expression of TLRs and MyD88 but a reduced expression of NF-*κ*B in U937 cells of the coinfection model in the presence of rapamycin, in comparison with the absence of an inhibitor. (b) The fold of changes of proteins of interest in (a) semiquantitatively determined by densitometric assay using ImageJ software from three independent experiments. (c) Concentrations of TNF-*α*, IL-10, and IL-6 in culture media determined by ELISA. An increased TNF-*α* was observed in the presence of mTOR inhibitor rapamycin. The A549 cell-mediated reduction of cytokines in the *Mtb* H37Rv-infected U937 cells was reversed in the presence of mTOR inhibitor rapamycin. Error bars represent the standard deviation (SD) from three independent experiments. Compared to noninfection (NI) control, ^∗^ *p* < 0.05 and ^∗∗^ *p* < 0.01; compared to the absence of LiCl, ^Δ^ *p* < 0.05 and ^ΔΔ^ *p* < 0.01. NI: noninfected control; AI: infection was performed on A549 cell alone; UI: infection was performed on U937 alone; CI: infection was performed on both A549 cells and U937 cells.

Next, seeking to investigate the function of PI3K/Akt/mTOR signaling in the epithelial cell-modulated immune responses in U937 cell-derived macrophage-like cells to *Mtb* infections, the TLR-mediated inflammatory responses of U937 macrophage-like cells were evaluated in the *Mtb* H37Rv-infected A549/U937 macrophage-like cell coculture model in the presence of mTOR inhibitor rapamycin (Rapa) at a final concentration of 100 nM (Santa Cruz, Santa Cruz, CA, USA). Immunoblotting assay showed that an exposure of A549/U937 cocultured cells to rapamycin altered the expression of *Mtb*-induced TLR-4 and TLR-6 proteins, despite that MyD88 was not significantly changed regardless of the presence of rapamycin (Figures 9(a) and 9(b)). The presence of rapamycin reduced the TLR-4 protein of U937 macrophage-like cells in the condition of *Mtb*-infected A549 cells alone but significantly elevated TLR-4 of U937 macrophage-like cells when both A549 and U937 cells were infected. However, the addition of rapamycin increased the expression of *Mtb*-induced TLR-6 expression but inhibited the expression of NF-*κ*B and mTOR in U937 macrophage-like cells of all three conditions of infection (Figure 9). As a consequence, the *Mtb*-induced production of cytokine IL-6 and IL-10 was also significantly reduced in the condition of *Mtb*-infected U937 cells alone with rapamycin (*p* < 0.01) (Figure 9). Surprisingly, the addition of rapamycin completely abolished (NF-*κ*B) or even elevated the A549 cell-attenuated *Mtb*-activated TLR signaling (TLR-4, TLR-6) and cytokine production (IL-6, IL-10) in U937 macrophage-like cells (Figure 9), suggesting a crucial role of PI3K/Akt/mTOR signaling in the modulation of epithelial cells to immune responses of macrophages in *Mtb* infections.

## 4. Discussions

The interactions with alveolar epithelial cells have been demonstrated to be one of the mechanisms modulating the phenotypic pattern of alveolar macrophages in both steady and pathological states [7, 10, 17–19]. In the present study, by using an air-liquid interface (ALI) coculture model generated with A549 epithelial cells and U937 monocyte-derived macrophage-like cells, the modulation of epithelial cells in *Mtb*-triggered TLR signaling activation in macrophages was explored. Results showed that a coinfection of *Mtb* H37Rv to A549 epithelial cells significantly attenuated the expression of TLR ligands and elements of the TLR signaling pathway in U937 macrophage-like cells to the pathogen infection, as compared with the infection of U937 cells alone. A mechanistic study using small molecule inhibitors of PI3K, mTOR, and GSK3*β* revealed that PI3K/Akt/mTOR signaling but not PI3K/Akt/GSK3*β* signaling played a major role in the epithelial cell-modulated *Mtb*-driven inflammatory responses of U937 macrophage-like cells. This study suggests that the *Mtb*-induced epithelial cell-derived signals (cytokines, chemokines, and/or costimulatory molecules) may be key immune modulators in TLR-mediated inflammatory responses of alveolar macrophages to an *Mtb* infection.

An increasing number of studies have demonstrated that pulmonary epithelial cells played an important role in host protective response to *Mtb* infection by either a direct response to the infection or an indirect effect via modulating immune responses of professional immune cells such as macrophages [2, 3, 13, 20]. Macrophages are the predominant “professional” immune cell population in the distal lung, in which the alveolar epithelia are physical and functional barriers to maintain the integrity of alveolar sac, as well as play a crucial role in the clearance of environmental insults by initiating and expanding local host defense mechanisms [5, 21, 22]. In this regard, alveolar epithelial cells are able to modulate the activity of macrophages and regulate both innate and adaptive immunities by secretion of cytokines and chemokines [23–25], herein providing a bridge to connect the innate and adaptive immune systems [2, 26]. In this context, both the soluble innate defense biomolecules secreted by alveolar epithelial cells and direct cell-cell contacts may facilitate the cellular communications between epithelial cells and macrophages, an important early step in the immune response of host cells to an *Mtb* infection [5, 27].

Indeed, there is a substantial evidence for interactions between alveolar epithelial cells and macrophages in alveolar sacs [2]. In this respect, alveolar epithelial cells are an important source of cytokines and mediators modulating the phenotype or activation of alveolar macrophages in response to an external stimulus, including the *Mtb* [2, 5, 10, 28]. A previous coculture study using Mono-Mac-6 Mphis (MM6-Mphis) macrophages and epithelial A549 cells demonstrated that a direct cell contact between epithelial cells and macrophages led a decreased intracellular growth of *Mtb* in infected macrophages, despite that the bacterial growth in A549 cells was not affected [29]. The cell contact-decreased bacterial replication was attributed to cytokines secreted by *Mtb*-infected epithelial cells, such as TNF-*α* and a granulocyte macrophage colony-stimulating factor (GMCSF) [29]. Similarly, a directly contacted coculture of monolayers of A549 cells and THP-1 monocytes showed the epithelial cell-induced CD54 (ICAM-1), HLA-DR, IL-6, and IL-8 in monocytes under an unstimulated state [6, 30]. Interestingly, a blocking of NF-*κ*B signaling in A549 cells by p65 subunit siRNA inhibited the production of IL-6 in THP-1 cells, suggesting that the unstimulated epithelial cell regulated NF-*κ*B-dependent immune responses in human macrophages [30]. However, the above cell contact-dependent epithelial cell-amplified immune responses could be abrogated in cocultured cells separated by filter inserts, implying that the cell-cell contact was important for the epithelial cell-enhanced activation of macrophages [6, 30].

In the present study, the immunoregulatory role of epithelial cells in macrophages was examined in a contact-independent coculture model of A549 cells and U937 monocyte-derived macrophage-like cells and the result showed that the *Mtb*-coinfected A549 epithelial cells displayed a potential to alleviated TLR-mediated inflammatory responses in U937 cells to an *Mtb* infection. This result was in agreement with the above finding in THP-1 cells, i.e., the epithelial cell-enhanced immune responses could be abrogated in the filter-separated coculture [6, 30], although the THP-1 cells and U937 cells were different types of monocytes. However, whether a directly contacted coculture of A549 cells and U937-derived macrophage-like cells also exhibited an augmented immune response in U937 macrophage-like cells needs further investigation. Of note, the epithelial cell-mediated attenuation of *Mtb*-induced TLR-mediated inflammatory responses in U937 macrophage-like cells was cell contact independent, suggesting that the contact-independent communication between alveolar epithelial cells reduced immune responses of macrophages to *Mtb* in an early stage of infections. Indeed, such an early immune interaction of epithelial cells and macrophages was recently confirmed in another study by using a coculture model of human primary bronchial epithelial cells (PBECs) and THP-1 monocytes, in which the epithelial cells were found to express the same transcriptomic pattern in response to *Mtb* infection to that of alveolar macrophages or THP-1 cells [28]. By using this novel model, the authors demonstrated that PBECs were inert to direct *Mtb*-infection and not potent responders in an IL-1*β*- and type I interferon- (IFN-) mediated *Mtb*-activated immune network. However, the immune responses of PBECs to *Mtb* could be activated by pathogen-infected alveolar macrophages or THP-1 monocytes [28]. The activation of epithelial cells subsequently led an increased expression of known and novel antimycobacterial peptides. In addition, the interactions between epithelial cells and macrophages could further shape the immunological environment of alveoli during an *Mtb* infection by promoting neutrophil influx [28]. This study revealed that the *Mtb*-infected macrophages could activate immune responses of epithelial cells to *Mtb*, suggesting that the alveolar epithelial cells were potent responders to infected alveolar macrophages, which in turn was able to complement and amplify responses of the alveolar microenvironment to eliminate *Mtb* infections [28].

By using a similar strategy to the above study, in the present study, the role of epithelial cells in inflammatory responses of *Mtb*-infected macrophages was interrogated using the coculture of the air-liquid interface (ALI) state of A549 epithelial cells and U937 monocyte-derived macrophage-like cells. Despite A549 cells being cultured in an ALI state displayed properties of alveolar epithelial type I and type II cells, as determined by the expression of cell-type-specific markers, the biology and function between cell lines (A549 cells and U937 cells) and primary cells (epithelial cells and macrophages) were not identical in a great extent [31]. Nonetheless, an emerging role of *Mtb*-infected A549 epithelial cells in modulating TLR-mediated inflammation of *Mtb*-infected U937 macrophage-like cells was observed in the A549 cell/U937 macrophage-like cell ALI culture model, i.e., the *Mtb*-activated epithelial cells were able to attenuate the TLR-mediated inflammation of *Mtb*-infected macrophages with less cytokine secretion in the cell contact-independent coculture model. This finding differed from the function of *Mtb*-infected macrophages in epithelial cells to *Mtb* infection, i.e., the *Mtb*-infected macrophages complemented or amplified immune responses of epithelial cells to the infection [28]. Interestingly, the *Mtb*-activated TLR signaling and cytokines of U937 cells were significantly reduced when A549 cells were coinfected with Mtb H37Rv, in comparison with the U937 cell infection alone. However, the TLR signaling cascade was activated in A549 cells upon the Mtb infection regardless of A549 cells and/or U937 macrophage-like cells. In this regard, the infection of U937 cells alone showed the most robust induction of transcripts of TLR signaling elements and cytokines in A549 cells. Equally noteworthy, the function of uninfected epithelial cells in the macrophage response to infection and the impact of infected macrophages to *Mtb*-infected epithelial cells have not been extensively investigated, which was a limitation of this study. Nonetheless, these results clearly indicated an important role of *Mtb*-activated A549 epithelial cells in modulating TLR-mediated inflammation of U937 monocyte-derived macrophage-like cells to the infection in a contact-independent manner. Such a negative modulation of epithelial cells to macrophages might be crucial for maintaining the immune homeostasis and balance between a proinflammatory and an immunoregulatory state in alveolar microenvironment, thereby protecting host cells from injury caused by excessive inflammatory responses or allowing the establishment of infection [32]. Therefore, the cellular communication between epithelial cells and macrophages may play a key role in the homeostasis of the alveolar environment to infection of *Mbt*. In this context, the *Mtb*-activated macrophages could potentiate the immune responses of epithelial cells to the pathogen [28]. Vice versa, interestingly, the *Mtb*-infected epithelial cells were able to alleviate the inflammation of macrophages to *Mtb*, suggesting a protective role of epithelial cells in the early stage after infection [28].

Mechanistically, a growing body of evidence suggests that the PI3K pathway is involved in TLR signaling-mediated inflammation and the release of cytokines of macrophages [33, 34]. An inhibition of PI3K signaling led to an amelioration of immune response and a suppression of the secretion of proinflammatory cytokines in immune cells [35]. In line with these findings, the *Mtb*-activated TLR signaling and proinflammatory cytokines of U937 monocyte-derived macrophage-like cells could be abolished by an addition of PI3K inhibitor LY294002. More interestingly, the *Mtb*-infected A549 epithelial cell-reduced expression of TLR signaling elements and cytokines in U937-derived cells to *Mtb* was reversed or lost in the presence of PI3K inhibitor LY294002, suggesting that the epithelial cell-modulated inflammatory responses of macrophages to *Mtb* were mediated by the PI3K signaling pathway.

In order to further dissect the underlying mechanism of PI3K signaling in immune regulation of epithelial cells to macrophages in response to *Mtb* infection, the involvement of downstream of PI3K signaling cascade, the PI3K/Akt/GSK3*β* and PI3K/Akt/mTOR pathways were explored. It is well characterized that PI3K/Akt signaling pathway regulated a cell context-dependent inactivation of GSK3*β* or activation of mTOR signaling [36, 37]. In this regard, PI3K/Akt/GSK3*β* signaling showed an ability to modulate the production of inflammatory cytokines in an NF-*κ*B-dependent manner [38–40], while the PI3K/Akt/mTOR pathway was a major cellular signaling pivot with a large spectrum of cellular functions in response to extracellular stimuli [41–43].

To further elucidate the roles of PI3K/Akt/GSK3*β* in the epithelial modulation of inflammatory responses in U937 macrophage-like cells, the GSK3*β* signaling was blocked in the *Mtb*-infected coculture of epithelial cell/macrophage by the addition of GSK3*β* inhibitor LiCl. A previous study has demonstrated that *Mycobacterium bovis* bacilli Calmette-Guerin (BCG) induced the phosphorylation of GSK3 by PI3K/Akt in the signaling pathway downstream of TLR2 and TLR4 in primary epithelial cells. The BCG-induced GSK3 inhibition could not affect the proinflammatory NF-*κ*B and led to the production of anti-inflammatory IL-10 and IL-22 [44]. In consistency with this finding, an inhibition of GSK3*β* with LiCl led to a moderate increase of NF-*κ*B in U937 macrophage-like cells to *Mtb* H37Rv (Figure 7(a)). However, the addition of LiCl did not alter the activity of TLR/MyD88 signaling and the production proinflammatory cytokines IL-6 and TNF-*α* but the secretion of anti-inflammatory cytokine IL-10 was increased. Unexpectedly, the GSK3*β* inhibitor LiCl failed to reverse the *Mtb*-infected epithelial cell-reduced expression of TLR signaling elements and cytokines in U937-derived cells to Mtb, suggesting that the immune modulatory role of epithelial cells in macrophages might not be mediated by the PI3K/Akt/GSK3*β* signaling.

The role of PI3K/Akt/mTOR signaling in the regulation of inflammations has been well documented. This cellular signaling has been suggested to have an important implication in the pathogenesis of tuberculosis. Indeed, the PI3K/Akt/mTOR signaling pathway was impaired in the T lymphocytes of patients with active tuberculosis [16]. It has been demonstrated that BCG could activate phosphorylation of Akt in host cells. Intriguingly, the inhibition of PI3K, Akt, or mTOR activity led to a reduction of intracellular infection in mammary epithelial cancer MCF-7 cells to BCG, indicating a critical role of the PI3K/Akt/mTOR pathway in the immune response of mycobacterial infection [45]. Moreover, pharmacological inhibitions of PI3K/AKT/mTOR signaling led to a drastic reduction of the production of proinflammatory cytokines in monocytes and macrophages [46] and reversed the LPS-stimulated increase of TLR4 expression and decrease of the peroxisome proliferator-activated receptor gamma (PPAR-gamma) level in human endothelial cells [47]. Consistently, the inhibition of PI3K or mTOR with LY294002 or rapamycin (Rapa) led to a dramatically reduced expression of TLR signaling elements and cytokine production in U937 macrophage-like cells to *Mtb* infection in the present study. Even more importantly, a blocking of the PI3K/Akt/mTOR pathway with LY294002 or Rapa restored the *Mtb*-infected epithelial cell-alleviated inflammatory responses in macrophages to the pathogen, together with the inability of GSK3*β* inhibitor LiCl to reverse the epithelial cell-mediated attenuation of inflammation in U937 macrophage-like cells to *Mtb*. These results suggest that the epithelial cell-alleviated inflammatory response of macrophages to *Mtb* was mainly mediated by the PI3K/Akt/mTOR signaling pathway. This finding also provides a fine-tuned molecular mechanism underlying the interaction and communication between epithelial cells and macrophages for maintaining an immune homeostasis of microenvironment alveolar sac in response to *Mtb* infections.

**Figure 10:**
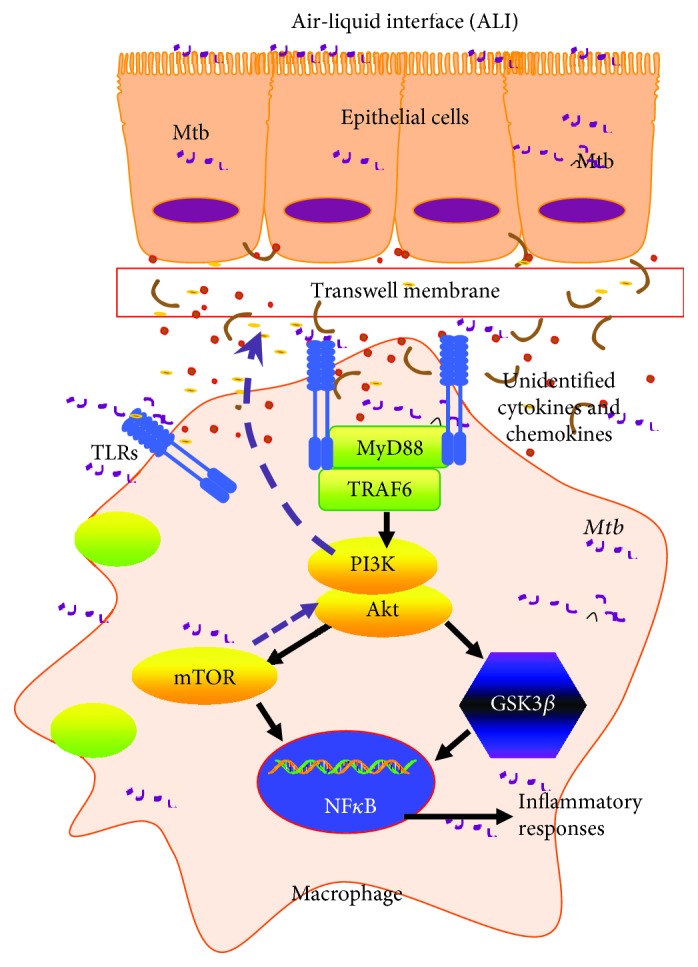
Scheme showing a possible mechanism of the alleviated TLR-mediated inflammatory responses of macrophage U937 cells to the mycobacterial infection by coinfected A549 epithelial cells. In a coinfected condition, the undefined signaling from H37Rv mycobacteria-infected epithelial cells could reduce TLR-mediated inflammation of macrophages via the PI3K/Akt/mTOR signaling axis, but not the PI3K/Akt/GSK3*β* pathway. Upon a coinfection of epithelial cells and macrophages to *Mtb*, the inflammatory responses in both host cell types were triggered in alveoli. In order to maintain the homeostasis of the alveolar microenvironment, the *Mtb* infection-induced epithelial cells subsequently alleviated the inflammation of the alveolar environment to secret soluble cytokines or mediators, which in turn inhibited the TLR-mediated inflammatory responses in macrophages to *Mtb* via the PI3K/Akt/mTOR pathway.

Collectively, by using an air-liquid interface (ALI) coculture model of human alveolar epithelial A549 cells and mononuclear U937 cell-derived macrophage-like cells, a coinfection of epithelial cells and macrophages to *Mtb* significantly inhibited the TLR-mediated cytokine production in macrophages, indicating an important role of epithelial cells in maintaining the homeostasis of the alveolar microenvironment. Mechanistically, the epithelial cell-ameliorated inflammatory response in macrophages to *Mtb* was mainly mediated by the PI3K/Akt/mTOR pathway rather than PI3K/Akt/GSK3*β* pathway (Figure 10). However, there were several limitations in this study; firstly, the A549 cell and U937 monocyte cell line rather than primary epithelial cells and macrophages were used in the coculture model; secondly, in primary cells, pharmacological inhibitors instead of genetic strategies such as gene knockdown or knockoff were employed for inhibitions of signaling cascades and the expression of TLR signaling elements and cytokine production in A549 epithelial cells have not been systematically analyzed. Nevertheless, this study provides an insight into mechanisms underpinning the cell contact-independent cellular communication between epithelial cells and macrophages in *Mtb* infections.

## Figures and Tables

**Table 1:** The real-time qRT-PCR primers used in this study.

Primers	GenBank number	Sequence (5′-3′)	Tm	Size (bp)
*β*-Actin	NM_001101.3	F: TTCGTGGATGCCACAGGACT	61°C	212
		R: GGGAAATCGTGCGTGACATT		
IL-1*β*	NM_000576.2	F: ATGACCTGAGCACCTTCTTTC	62°C	82
		R: TGCACATAAGCCTCGTTATCC		
IL-2	NM_000586.3	F: CTCACCAGGATGCTCACATTTA	60°C	240
		R: TACAATGGTTGCTGTCTCATCA		
IL-6	NM_000600.3	F: GACAGCCACTCACCTCTTCAG	59°C	172
		R: CATCCATCTTTTTCAGCCATC		
IL-8	NM_000584.3	F: TTGCCAAGGAGTGCTAAAGAA	61°C	215
		R: GCCCTCTTCAAAAACTTCTCC		
IL-10	NM_000572.2	F: TTTAAGGGTTACCTGGGTTGC	60°C	98
		R: TTGATGTCTGGGTCTTGGTTC		
IL-12*β*	NM_000882.3	F: AGCAGTGAGGTCTTAGGCTCTG	60°C	179
		R: TTGGGTTCTTTCTGGTCCTTTA		
TNF-*α*	NM_000594.2	F: TAGCCCATGTTGTAGCAAACC	63°C	136
		R: ATGAGGTACAGGCCCTCTGAT		
MyD88	NM_002468.4	F: CCAGTTTGTGCAGGAGATGA	61°C	288
		R: AGGATGCTGGGGAACTCTTT		
TLR2	NM_003264.3	F: ATGCTGCCATTCTCATTCTTCT	60°C	101
		R: CTCCAGGTAGGTCTTGGTGTTC		
TLR4	NM_138554.3	F: CTTCTCAACCAAGAACCTGGAC	60°C	158
		R: TAGAGAGGTGGCTTAGGCTCTG		
TLR6	NM_006068.4	F: CTACCGCTGAAAACCAAAGTCT	60°C	322
		R: ACTCACAATAGGATGGCAGGAT		
TLR8	NM_138636.4	F: CCTGGCTCACCATTTGTTTTA	60°C	198
		R: TTTTTGTCTCGGCTCTCTTCA		
NF-*κ*B	NM_002502.5	F: AGGAGAGGATGAAGGAGTTGTG	62°C	218
		R: CCAGAGTAGCCCAGTTTTGTC		
TRAF6	NM_145803.2	F: TAGCCCTGGATTCTACACTGG	63°C	215
		R: CTTCGTGGTTTTGCCTTACAG		

## Data Availability

The data used to support the findings of this study are included within the article.

## Supplementary Materials

Supplementary Materials Supplementary Figure 1: a coinfection of *Mtb* H37Rv to A549 epithelial cells and U937 cells reduced the expression of TLR signaling elements in A549 cells. The coculture model of A549/U937 macrophage-like cells was infected with H37Rv *Mycobacteria* from the upper chamber (A549 cells, AI), lower chamber (U937 cells, UI), or both chambers (A549 and U937 cells, CI) at a MOI of 3 for 18 h before the A549 cells were harvested for analysis by RT-PCR assay. (A–G) Inductions of indicated transcripts in A549 cells in cocultures infected with H37Rv in different conditions. (A) Fold of changes of TLR-2 transcripts over the noninfected cells; (B) fold of changes of TLR-4 transcripts over the noninfected cells; (C) fold of changes of TLR-6 transcripts over the noninfected cells; (D) fold of changes of TLR-8 transcripts over the noninfected cells; (E) fold of changes of MyD88 transcripts over the noninfected cells; (F) fold of changes of TRAF6 transcripts over the noninfected cells; (G) fold of changes of NF-*κ*B transcripts over the noninfected cells. Error bars represent the standard deviation (SD) from three independent experiments. Compared to noninfection (NI) control, ^∗∗^ *p* < 0.01; compared to infection of U937 cell alone, ^ΔΔ^ *p* < 0.01. NI: noninfected control; AI: infection was performed on A549 cell alone; UI: infection was performed on macrophage-like cells alone; CI: infection was performed on both A549 cells and U937 cells. Supplementary Figure 2: a coinfection of *Mtb* H37Rv to A549 epithelial cells and U937 cells reduced the expression of cytokines in A549 cells. The coculture model of A549/U937 macrophage-like cells was infected with H37Rv *Mycobacteria* from the upper chamber (A549 cells, AI), lower chamber (U937 cells, UI), or both chambers (A549 and U937 cells, CI) at a MOI of 3 for 18 h before the A549 cells were harvested for analysis by RT-PCR assay. (A–G) Inductions of indicated transcripts in A549 cells in cocultures infected with H37Rv in different conditions. (A) Fold of changes of IL-1*β* transcripts over the noninfected cells; (B) fold of changes of IL-2 transcripts over the noninfected cells; (C) fold of changes of IL-6 transcripts over the noninfected cells; (D) fold of changes of IL-8 transcripts over the non-infected cells; (E) fold of changes of IL-10 transcripts over the noninfected cells; (F) fold of changes of IL-12*β* transcripts over the noninfected cells; (G) fold of changes of TNF-*α* transcripts over the non-infected cells. Error bars represent the standard deviation (SD) from three independent experiments. Compared to noninfection (NI) control, ^∗∗^ *p* < 0.01; compared to infection of U937 macrophage-like cells alone, ^ΔΔ^ *p* < 0.01. NI: noninfected control; AI: infection was performed on A549 cell alone; UI: infection was performed on U937 alone; CI: infection was performed on both A549 cells and U937 cells.
