# Erratum to “Epithelial Cells Attenuate Toll-Like Receptor-Mediated Inflammatory Responses in Monocyte-Derived Macrophage-Like Cells to *Mycobacterium tuberculosis* by Modulating the PI3K/Akt/mTOR Signaling Pathway”

**DOI:** 10.1155/2021/3710790

**Published:** 2021-03-13

**Authors:** Yi Yang, Yingfei Sun, Jinrui Xu, Kangda Bao, Meihui Luo, Xiaoming Liu, Yujiong Wang

**Affiliations:** ^1^Key Laboratory of Ministry of Education for Conservation and Utilization of Special Biological Resources in Western China, Ningxia University, Yinchuan, Ningxia 750021, China; ^2^College of Life Science, Ningxia University, Yinchuan, Ningxia 750021, China

In the article titled “Epithelial Cells Attenuate Toll-Like Receptor-Mediated Inflammatory Responses in Monocyte-Derived Macrophage-Like Cells to *Mycobacterium tuberculosis* by Modulating the PI3K/Akt/mTOR Signaling Pathway” [1], there was an error in Figure 9. The band of “TLR5, NF*κ*6, mTOR, and pmTOR” is identical to the band of “TLR4” shown in the “without inhibitor.” The corrected figure is shown below as Figure 9.

## Figures and Tables

**Figure 1:**
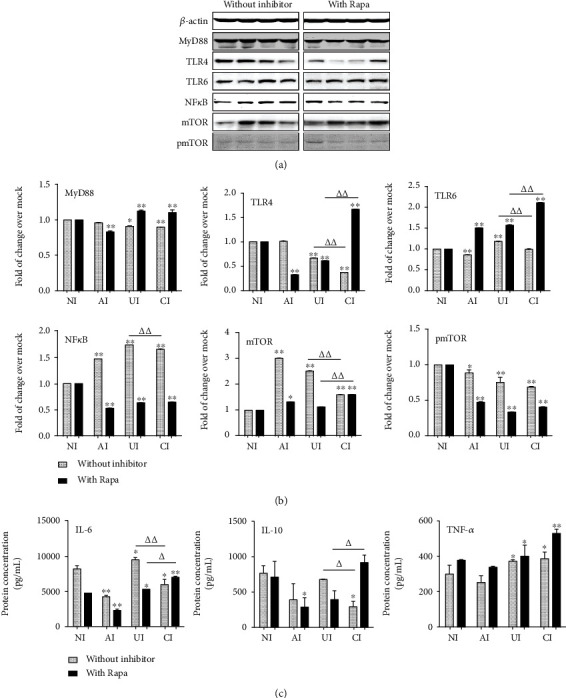
Impact of mTOR on the expression of TLR ligands and elements of the TLR signaling pathway in U937 cells to H37Rv infection in the A549 cell coculture model. In the presence or absence of mTOR inhibitor rapamycin, the coculture model of A549/U937 cells was infected with H37Rv mycobacteria from the upper chamber (A549 cells, AI), lower chamber (U937 cells, UI), or both chambers (A549 and U937 cells, CI) at a MOI of 3 for 18 h before the culture medium, and U937 cells were harvested for analysis. (a) Representative blots of immunoblotting assay for indicated components of TLR signaling cascade showed an increased expression of TLRs and MyD88 but a reduced expression of NF*κ*B in U937 cells of the coinfection model in the presence of rapamycin, in comparison with the absence of an inhibitor. (b) The fold of changes of proteins of interest is (a) semiquantitatively determined by densitometric assay using ImageJ software from three independent experiments. (c) Concentrations of TNF-*α*, IL-10, and IL-6 in culture media determined by ELISA. An increased TNF-*α* was observed in the presence of mTOR inhibitor rapamycin. The A549 cell-mediated reduction of cytokines in the Mtb H37Rv-infected U937 cells was reversed in the presence of mTOR inhibitor rapamycin. Error bars represent the standard deviation (SD) from three independent experiments. Compared to noninfection (NI) control, ^∗^*p* < 0 05 and ^∗∗^*p* < 0 01; compared to the absence of LiCl, *^Δ^p* < 0.05 and *^ΔΔ^p* < 0.01. NI: noninfected control; AI: infection was performed on A549 cell alone; UI: infection was performed on U937 alone; CI: infection was performed on both A549 cells and U937 cells.
