# Supplementary material for: Epithelial Cells Attenuate Toll-Like Receptor-Mediated Inflammatory Responses in Monocyte-Derived Macrophage-Like Cells to Mycobacterium tuberculosis by Modulating the PI3K/Akt/mTOR Signaling Pathway
Source: Mediators Inflamm. 2018 Sep 26;2018:3685948. doi: 10.1155/2018/3685948 (PMC6178170; doi:10.1155/2018/3685948)
Supplement: Supplementary Materials — Supplementary Figure 1: a coinfection of Mtb H37Rv to A549 epithelial cells and U937 cells reduced the expression of TLR signaling elements in A549 cells. The coculture model of A549/U937 macrophage-like cells was infected with H37Rv Mycobacteria from the upper chamber (A549 cells, AI), lower chamber (U937 cells, UI), or both chambers (A549 and U937 cells, CI) at a MOI of 3 for 18 h before the A549 cells were harvested for analysis by RT-PCR assay. (A–G) Inductions of indicated transcripts in A549 cells in cocultures infected with H37Rv in different conditions. (A) Fold of changes of TLR-2 transcripts over the noninfected cells; (B) fold of changes of TLR-4 transcripts over the noninfected cells; (C) fold of changes of TLR-6 transcripts over the noninfected cells; (D) fold of changes of TLR-8 transcripts over the noninfected cells; (E) fold of changes of MyD88 transcripts over the noninfected cells; (F) fold of changes of TRAF6 transcripts over the noninfected cells; (G) fold of changes of NF-κB transcripts over the noninfected cells. Error bars represent the standard deviation (SD) from three independent experiments. Compared to noninfection (NI) control, ∗∗ p < 0.01; compared to infection of U937 cell alone, ΔΔ p < 0.01. NI: noninfected control; AI: infection was performed on A549 cell alone; UI: infection was performed on macrophage-like cells alone; CI: infection was performed on both A549 cells and U937 cells. Supplementary Figure 2: a coinfection of Mtb H37Rv to A549 epithelial cells and U937 cells reduced the expression of cytokines in A549 cells. The coculture model of A549/U937 macrophage-like cells was infected with H37Rv Mycobacteria from the upper chamber (A549 cells, AI), lower chamber (U937 cells, UI), or both chambers (A549 and U937 cells, CI) at a MOI of 3 for 18 h before the A549 cells were harvested for analysis by RT-PCR assay. (A–G) Inductions of indicated transcripts in A549 cells in cocultures infected with H37Rv in different conditions. (A) Fo [file 3685948.f1.doc]

**Supplementary data**

**Supplementary Fig. 1**


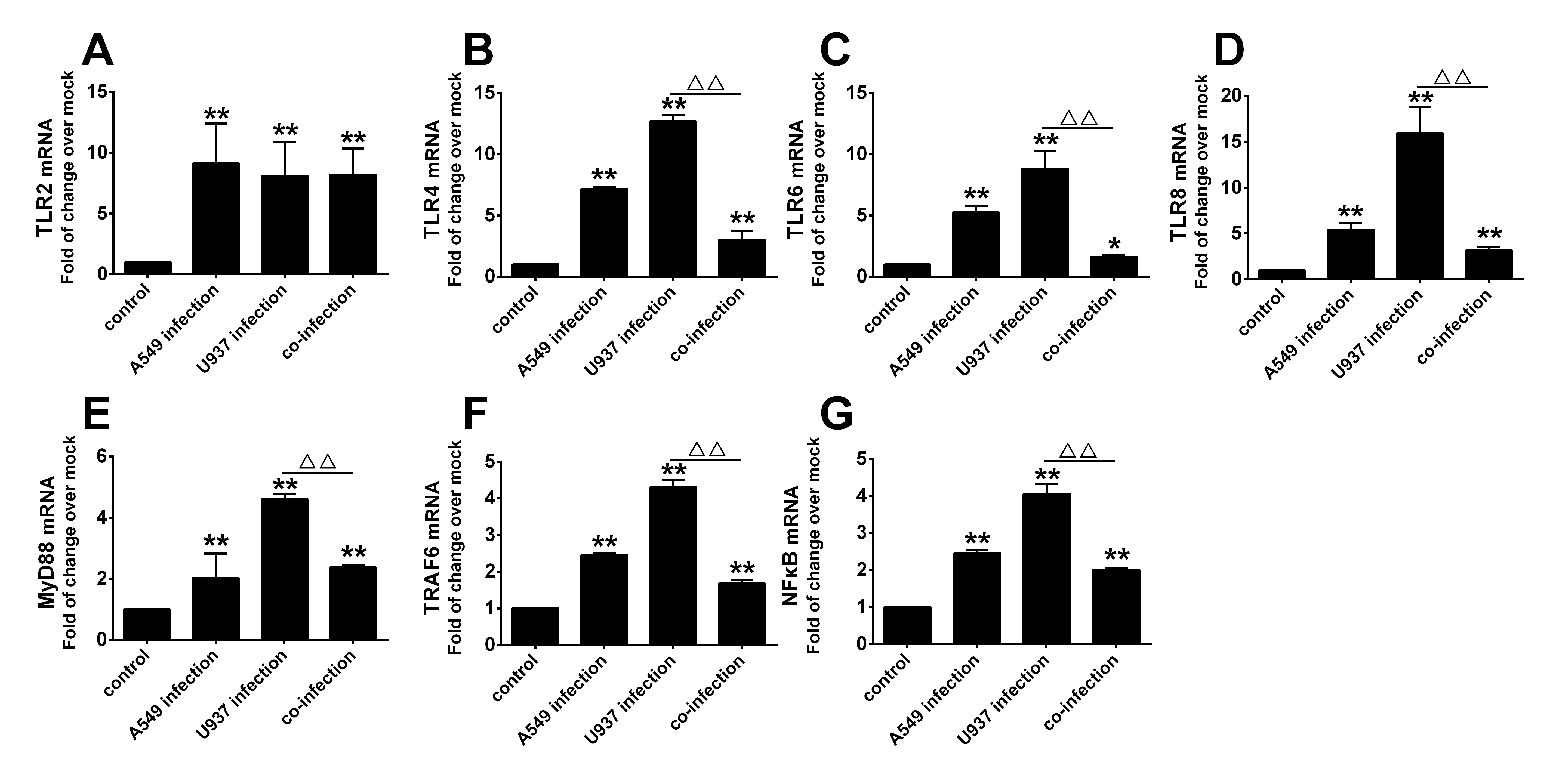


**Supplementary Figure 1.** **A Co-infection of** ***Mtb* H37Rv to A549 epithelial cells and U937 cells reduced the expression of TLR signaling elements in A549 cells**. The co-culture model of A549/U937 macrophage-like cells was infected with H37Rv *Mycobacteria* from upper chamber (A549 cells, AI), lower chamber (U937 cells, UI) or both chambers (A549 and U937 cells, CI) at MOI of 3 for 18 h before the A549 cells were harvested for analysis by a RT-PCR assay. (A-G) Inductions of indicated transcripts in A549 cells in co-cultures infected with H37Rv in different conditions. (A) Fold of changes of TLR-2 transcripts over the non-infected cells; (B) Fold of changes of TLR-4 transcripts over the non-infected cells; (C) Fold of changes of TLR-6 transcripts over the non-infected cells; (D) Fold of changes of TLR-8 transcripts over the non-infected cells; (E) Fold of changes of MyD88 transcripts over the non-infected cells; (F) Fold of changes of TRAF6 transcripts over the non-infected cells; (G) Fold of changes of NF-κB transcripts over the non-infected cells. Error bars represent the standard deviation (SD) from three independent experiments. Compared to non-infection (NI) control, **: p<0.01; compared to infection of U937 cell alone, ΔΔ: p<0.01. NI, non-infected control; AI, infection was performed on A549 cell alone; UI, infection was performed on macrophage-like cells alone; CI, infection was performed on both A549 cells and U937 cells.

**Supplementary Fig. 2**


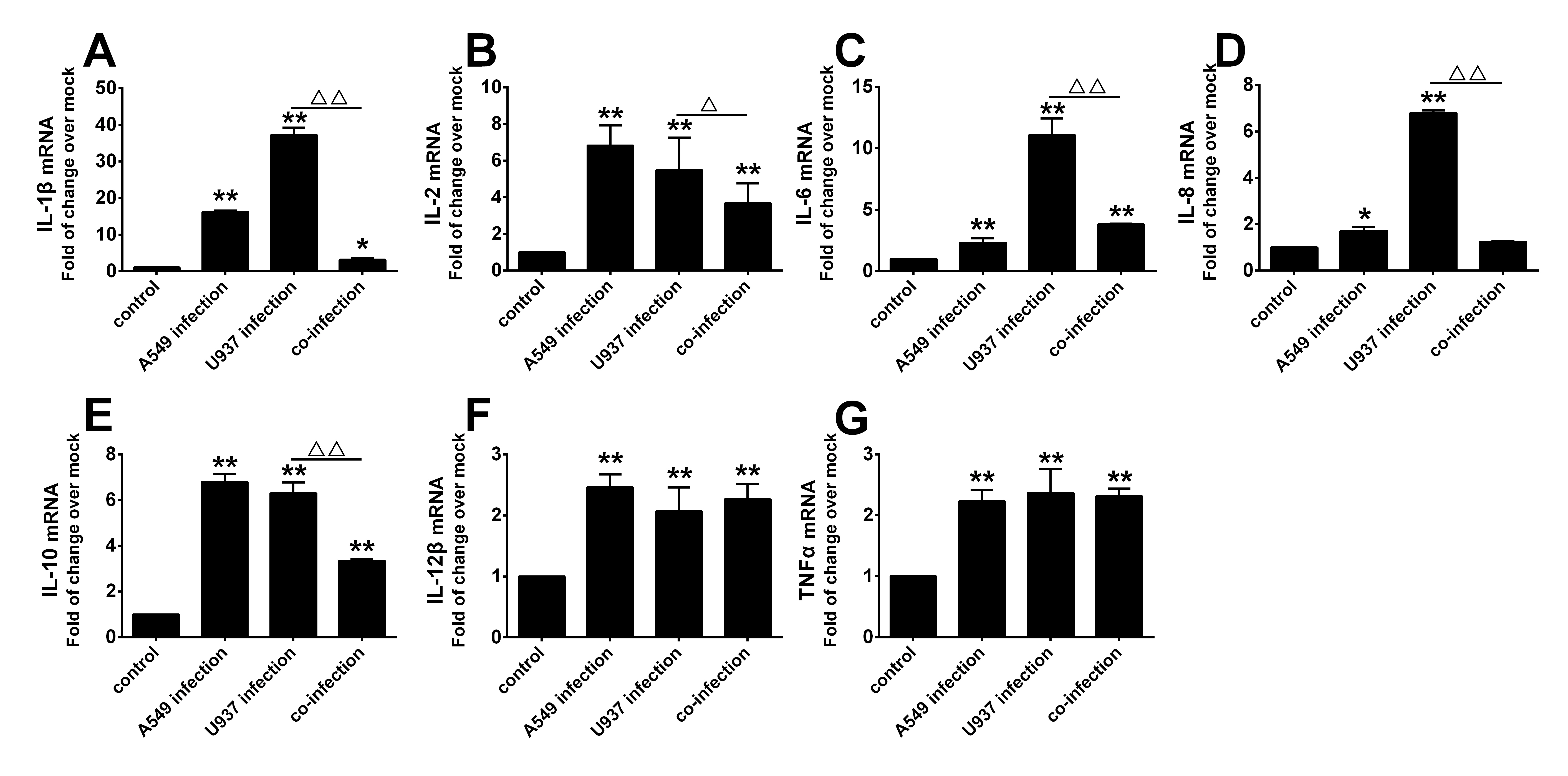


**Supplementary Figure 2.** **A Co-infection of** ***Mtb* H37Rv to A549 epithelial cells and U937 cells reduced the expression of cytokines in A549 cells**. The co-culture model of A549/U937 macrophage-like cells was infected with H37Rv *Mycobacteria* from upper chamber (A549 cells, AI), lower chamber (U937 cells, UI) or both chambers (A549 and U937 cells, CI) at MOI of 3 for 18 h before the A549 cells were harvested for analysis by a RT-PCR assay. (A-G) Inductions of indicated transcripts in A549 cells in co-cultures infected with H37Rv in different conditions. (A) Fold of changes of IL-1β transcripts over the non-infected cells; (B) Fold of changes of IL-2 transcripts over the non-infected cells; (C) Fold of changes of IL-6 transcripts over the non-infected cells; (D) Fold of changes of IL-8 transcripts over the non-infected cells; (E) Fold of changes of IL-10 transcripts over the non-infected cells; (F) Fold of changes of IL-12β transcripts over the non-infected cells; (G) Fold of changes of TNF-α transcripts over the non-infected cells. Error bars represent the standard deviation (SD) from three independent experiments. Compared to non-infection (NI) control, **: p<0.01; compared to infection of U937 macrophage-like cells alone, ΔΔ: p<0.01. NI, non-infected control; AI, infection was performed on A549 cell alone; UI, infection was performed on U937 alone; CI, infection was performed on both A549 cells and U937 cells.
